# TD/GC–MS analysis of volatile markers emitted from mono- and co-cultures of *Enterobacter cloacae* and *Pseudomonas aeruginosa* in artificial sputum

**DOI:** 10.1007/s11306-018-1357-5

**Published:** 2018-04-26

**Authors:** Oluwasola Lawal, Hugo Knobel, Hans Weda, Tamara M. E. Nijsen, Royston Goodacre, Stephen J. Fowler, the BreathDx consortium, Waqar M. Ahmed, Antonio Artigas, J. Bannard-Smith, Lieuwe D. J. Bos, Marta Camprubi, Luis Coelho, Paul Dark, Alan Davie, Emili Diaz, Gemma Goma, Timothy Felton, Stephen J. Fowler, Royston Goodacre, Hugo Knobel, Oluwasola Lawal, Jan-Hendrik Leopold, Tamara M. E. Nijsen, Pouline M. P. van Oort, Pedro Povoa, Craig Portsmouth, Nicholas J. W. Rattray, Guus Rijnders, Marcus J. Schultz, Ruud Steenwelle, Peter J. Sterk, Jordi Valles, Fred Verhoeckx, Anton Vink, Hans Weda, Iain R. White, Tineke Winters, Tetyana Zakharkina

**Affiliations:** 1 Division of Infection, Immunity and Respiratory Medicine, School of Biological Sciences, Faculty of Biology, Medicine and Health, The University of Manchester, Manchester, UK; 2 Philips Research, Royal Philips B.V., Eindhoven, The Netherlands; 3 Philips Innovation Labs, Philips Lighting, Eindhoven, The Netherlands; 4 School of Chemistry, Manchester Institute of Biotechnology, University of Manchester, Manchester, UK; 5 Manchester Academic Health Science Centre, The University of Manchester and Manchester University NHS Foundation Trust, Manchester, UK

**Keywords:** Bacteria, *Enterobacter cloacae*, Gas Chromatography-Mass Spectrometry, Infection, *Pseudomonas aeruginosa*, Volatile organic compounds

## Abstract

**Introduction:**

Infections such as ventilator-associated pneumonia (VAP) can be caused by one or more pathogens. Current methods for identifying these pathogenic microbes often require invasive sampling, and can be time consuming, due to the requirement for prolonged cultural enrichment along with selective and differential plating steps. This results in delays in diagnosis which in such critically ill patients can have potentially life-threatening consequences. Therefore, a non-invasive and timely diagnostic method is required. Detection of microbial volatile organic compounds (VOCs) in exhaled breath is proposed as an alternative method for identifying these pathogens and may distinguish between mono- and poly-microbial infections.

**Objectives:**

To investigate volatile metabolites that discriminate between bacterial mono- and co-cultures.

**Methods:**

VAP-associated pathogens *Enterobacter cloacae* and *Pseudomonas aeruginosa* were cultured individually and together in artificial sputum medium for 24 h and their headspace was analysed for potential discriminatory VOCs by thermal desorption gas chromatography–mass spectrometry.

**Results:**

Of the 70 VOCs putatively identified, 23 were found to significantly increase during bacterial culture (i.e. likely to be released during metabolism) and 13 decreased (i.e. likely consumed during metabolism). The other VOCs showed no transformation (similar concentrations observed as in the medium). Bacteria-specific VOCs including 2-methyl-1-propanol, 2-phenylethanol, and 3-methyl-1-butanol were observed in the headspace of axenic cultures of *E. cloacae*, and methyl 2-ethylhexanoate in the headspace of *P. aeruginosa* cultures which is novel to this investigation. Previously reported VOCs 1-undecene and pyrrole were also detected. The metabolites 2-methylbutyl acetate and methyl 2-methylbutyrate, which are reported to exhibit antimicrobial activity, were elevated in co-culture only.

**Conclusion:**

The observed VOCs were able to differentiate axenic and co-cultures. Validation of these markers in exhaled breath specimens could prove useful for timely pathogen identification and infection type diagnosis.

**Electronic supplementary material:**

The online version of this article (10.1007/s11306-018-1357-5) contains supplementary material, which is available to authorized users.

## Introduction

Lower respiratory tract infections such as pneumonia occur frequently in patients in intensive care units (ICUs), and can be mono- or poly-microbial (Kalanuria et al. 2014; Ferrer et al. 2015). Patients are generally first administered empirical antibiotics while identity of the causal pathogen(s) is confirmed. Full microbial identification can take up to 7 days with the potential for interim inappropriate antibiotic regimens, and this lack of appropriate antibiotic therapy guidance can lead to poor clinical outcomes (due to lack of efficacy), increased length of stay, and unnecessary antibiotic resistance (Combes et al. 2002; Ferrer et al. 2015; Mietto et al. 2013). In addition to being time consuming, the procedure for obtaining biological specimens from patients is often invasive. Thus, early and accurate detection of infection is a critical ambition for optimised patient management and antimicrobial stewardship on the ICU.

The analysis of VOCs in exhaled breath is proposed as a potential alternative diagnostic tool (Phillips 1992; Lourenco and Turner 2014; Ahmed et al. 2017; Lawal et al. 2017a). It is postulated that VOCs from causal pathogens of infection are exhaled and thus provides potential for early non-invasive detection. Bacterial VOCs may be investigated by means of headspace analysis of in vitro cultures. These VOCs are commonly ‘trapped’ on thermal desorption tubes and analysed by gas chromatography–mass spectrometry (GC–MS) (Boots et al. 2014; Neerincx et al. 2016; Filipiak et al. 2012). Other pre-concentration methods such as solid phase microextraction (Tait et al. 2014; Shestivska et al. 2011) and needle trap devices (Zscheppank et al. 2014) have been described. Online analysis which does not require prior sample enrichment has also been reported (Allardyce et al. 2006).

Whilst there has been a wealth of information regarding VOCs from bacterial mono-cultures, there is limited information regarding volatile metabolites from microbial co-cultures. Neerincx et al. have reported VOCs from *P. aeruginosa* and *Aspergillus fumigatus* co-culture (2016) and Zhu et al. cultured the former pathogen with *Staphylococcus aureus* (Zhu et al. 2010). Microorganisms associated with poly-microbial infections have been reported in literature (Ferrer et al. 2015). A common combination includes a clinically relevant member of the *Enterobacteriaceae* family such as *Enterobacter cloacae*, and a non-fermenting Gram-negative rod-shaped bacterium for example *Pseudomonas aeruginosa* (Davin-Regli and Pages 2015; John et al. 1982). *E. cloacae* is commonly found in the lower gastrointestinal tract and antibiotic administration has been linked to its overgrowth resulting in translocation and pathogenicity (Park 2005). *P. aeruginosa* is ubiquitously present in soil and water and is an opportunistic pathogen which thrives when host defense mechanisms are impaired. To investigate potential discriminatory mono- and co-culture VOC markers, *E. cloacae* and *P. aeruginosa* were cultured separately and together in artificial sputum medium (ASM), in order to mimic the clinical diagnostic specimen. In addition, we have previously shown the influence of medium substrates on VOC profiles (Lawal et al. 2017b). The use of ASM culminated in the detection of diverse VOCs justifying its use in this study. VOCs in the headspace of bacterial cultures were trapped onto sorbent tubes and analysed by thermal desorption gas chromatography (TD–GC/MS). In addition, we seek to explore the growth relationship of both microbes. By performing this in vitro investigation, observed changes in VOC profile may aid in distinguishing between types of infections and may translate towards improved antibiotic therapy guidance.

## Materials and methods

### Media preparation

Tryptic soy agar (TSA), was made by combining tryptic soy broth (Oxoid, Basingstone, UK) and Agar bacteriological (Agar NO. 1, Oxoid) as specified by the manufacturer. Levine EMB agar (LEA) (Fluka Analytical, UK) was prepared according to manufacturer’s specification, and details for ASM preparation can be found in (Diraviam Dinesh 2010). Briefly to prepare 1 L of ASM, type II mucin (5 g; Sigma-Aldrich, Germany), salmon sperm DNA (4 g; Sigma-Aldrich, Germany), diethylenetriaminepentaacetic acid (5.9 mg; Sigma-Aldrich, Germany), sodium chloride (5 g; Sigma-Aldrich, Germany), potassium chloride (2.2 g; Sigma-Aldrich, Germany), Tris base (1.8 g; Sigma-Aldrich, Germany), egg yolk emulsion (5 mL; Oxoid), casamino acids (5 g; BD, Sparks, USA) were all dissolved in distilled water and subsequently autoclaved at 121 °C for 15 min.

### Bacterial culture

*E. cloacae*, DSM 30054 and *P. aeruginosa*, ATCC 10145 were used in this study. The strains were retrieved from glycerol frozen stock, sub-cultured twice on TSA plates to ensure purity and incubated overnight at 37 °C to obtain axenic colonies. Single colonies were subsequently transferred into 50 mL ASM in 250 mL Schott Duran glass bottles and incubated in an orbital shaker (innova®40 incubator shaker series, New Brunswick Scientific) at 37 °C with 200 rpm shaking to obtain overnight cultures. To investigate the growth relationship of the microorganisms and determine a time point for headspace sampling, approximately 10^6^ cells of each bacterium were added into the same glass bottle containing 50 mL ASM and incubated at 37 °C in an orbital shaker (innova®40) for 24 h. Corresponding mono-cultures were also established separately with the same amount of cells. To establish viable cell numbers, 1 mL aliquots were collected at 5, 8, 11, and 24 h from the distinct cultures and mixed with 9 mL physiological saline (0.9% NaCl). A classical microbiological plating approach was then performed which involved an initial dilution series and spreading of diluted homogeneous sample (100 µL) on LEA. The plates were then incubated at 37 °C for 24 h and the viable bacteria were noted as colony forming units (Stuart® colony counter, Barloworld Scientific Limited Stone, Staffordshire, UK). LEA is a selective and differential medium which allows the growth of Gram-negative bacterium and also produces different phenotypes for distinct microbes based on substrate conversions. In addition, 1 mL of bacterial cultures was collected for corresponding optical density (OD_600_) measurements (Eppendorf BioPhotometer plus, Eppendorf AG, Hamburg, Germany).

### Bacterial culture headspace sampling

Liquid mono- and co-cultures were prepared as described above. After incubation, and still positioned in the incubator to minimise condensation effects, the bottles containing the bacterial cultures were purged with dry nitrogen at a flow rate of 60 mL/min through customised caps (GL45 caps, Fischer scientific, UK). The headspace was simultaneously evacuated through a different outlet and again mixed with dry nitrogen introduced at a flow rate of 140 mL/min to lower the relative humidity below 100% at room temperature, thus minimising condensation, and was subsequently trapped onto custom made thermal desorption multi-bed sorbent tubes containing Tenax GR-Carbograph 5TD sorbents (Markes, Llantrisant, UK) using a pump at a flow rate of 200 mL/min for 6 min. Water vapour is known to interfere with the quantitative capture of VOCs hence the need to reduce its effects using dry nitrogen. The headspace was collected at 24 h as adequate growth was observed for both bacterium in co-culture. The sorbent tubes were purged with dry nitrogen again after sampling at a flow rate of 100 mL/min for 6 min.

### Gas chromatography–mass spectrometry (GC–MS) analysis

Internal calibration standards were spiked onto the sorbent tubes by dilution of gaseous calibration standards (10 ppmv acetone-*d*_6_, hexane-*d*_14_, toluene-*d*_8_, xylene-*d*_10_ in nitrogen, Air Products, Amsterdam, The Netherlands) using a custom-made dilution system. As a quality assurance procedure, empty and sorbent-only tubes are also analysed. Sorbent tubes were thermally desorbed at 225 °C (TDSA, Gerstel, Mülheim an der Ruhr, Germany) into the packed liner. Solvent venting mode was used to transfer the sample to the packed liner (filled with Tenax TA) held at − 55 °C which was subsequently heated to 280 °C to transfer the VOCs into the GC capillary column. A cold trap (CTS2, Gerstel, Mülheim an der Ruhr, Germany) was used to minimise band broadening (initial temperature − 150 °C, after 1.6 min heated to 220 °C). A capillary gas chromatograph (7890N GC, Agilent, Santa Clara, CA, USA) using a VF1-MS column (30 m × 0.25 mm, film thickness 1 µm, 100% dimethyl-polysiloxane, Varian Chrompack, Middelburg, The Netherlands) was used with the following temperature program: 30 °C, hold 3.5 min, ramp 5 °C/min to 50 °C, hold 0 min, ramp 10 °C/min to 90 °C, hold 0 min, ramp 15 °C/min to 130 °C, hold 0 min, ramp 30 °C/min to 180 °C, hold 0 min, ramp 40 °C/min to 280 °C, hold 1 min. A Time of Flight mass spectrometer (LECO Pegasus 4D system, LECO, St. Joseph, MI, USA) was used in electron ionization mode at 70 eV, with a scan range of *m*/*z* 29–400 Da, scanning rate 20 scans/s.

### Data processing and analysis

#### Data pre-processing

LECO Chromatof software (LECO, St. Joseph, MI, USA) was used to acquire, analyse, and manage GC–MS runs. The acquired chromatograms can be viewed using this software and mass spectra from individual peaks cross-referenced with National Institute of Standards and Technology library 14 (NIST, Gaithersburg, MD, USA) for putative identification purposes, and followed the metabolomics standards initiative (MSI) guidelines for metabolite identification (Sumner et al. 2007). Metabolites with a NIST match factor of ≥ 800 were investigated.

Raw data in manufacturer’s data format were converted into a netCDF format utilising the LECO Chromatof software. All statistical analyses were performed using the R software (version 3.4.2; R Core Team (2017)). The *xcms* package following the approach as outlined by Smith et al. (2006) was used to pre-process the netCDF files in R. The product of raw data pre-processing is a data file containing ion fragments, their corresponding *m*/*z*, retention times, and also integrated areas. Normalisation using the internal standard (IS), toluene-*d*_8_, was based on the 100 *m*/*z* parent ion.

### Univariate and multivariate analyses

For univariate analysis when applicable, the non-parametric Kruskal–Wallis test was performed. Games-Howell post-hoc test was then subsequently used to investigate statistically significant entities. A critical *α* = 0.05 value was used in tests. False discovery rate method was used to adjust the obtained *p*-values after multiple hypothesis testing.

Principal component–discriminant function analysis (PC–DFA) was used for multivariate exploration of data (Jombart et al. 2010; Goodacre et al. 1998). Briefly, the aim is to maximise the variance between groups and minimise variance within groups. Principal components (PCs) are the input variables to DFA therefore PCA on the X data (VOC profiles) precedes DFA; the R package *adegenet* was used for this analysis (Jombart 2008). For validation, the dataset was divided into 70% training and 30% test sets. A PC–DFA model was built using the training set and the test set was then projected into the subspace created by the training set to visualise the prediction of the test data on the basis of proximity to the training samples originating from the same bacterial groups.

## Results

### Appearance of *E. cloacae* and *P. aeruginosa* on Levine EMB agar

**Fig. 1:**
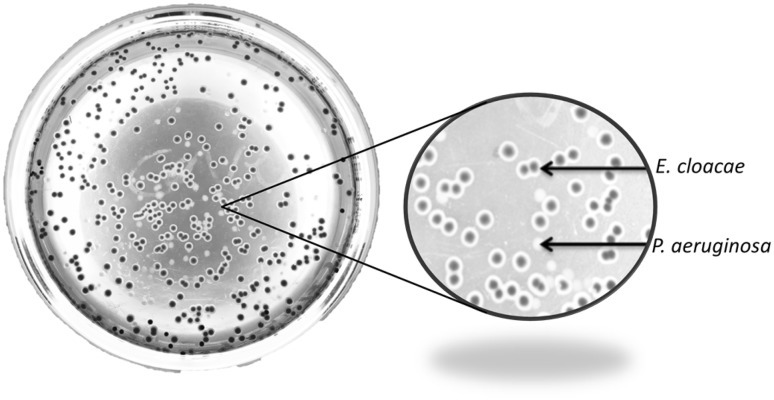
Phenotypes of *E. cloacae* (dark pink) and *P. aeruginosa* (white/colourless) colonies on selective and differential medium Levine EMB agar

Levine EMB agar was used as a selective and differential medium to distinguish between the bacteria species under investigation. *E. cloacae* appeared to develop dark pink colonies and colourless/white colonies were observed for *P. aeruginosa* (Fig. 1).

### *E. cloacae* and *P. aeruginosa* growth curves in mono- *versus* co-cultures

**Fig. 2:**
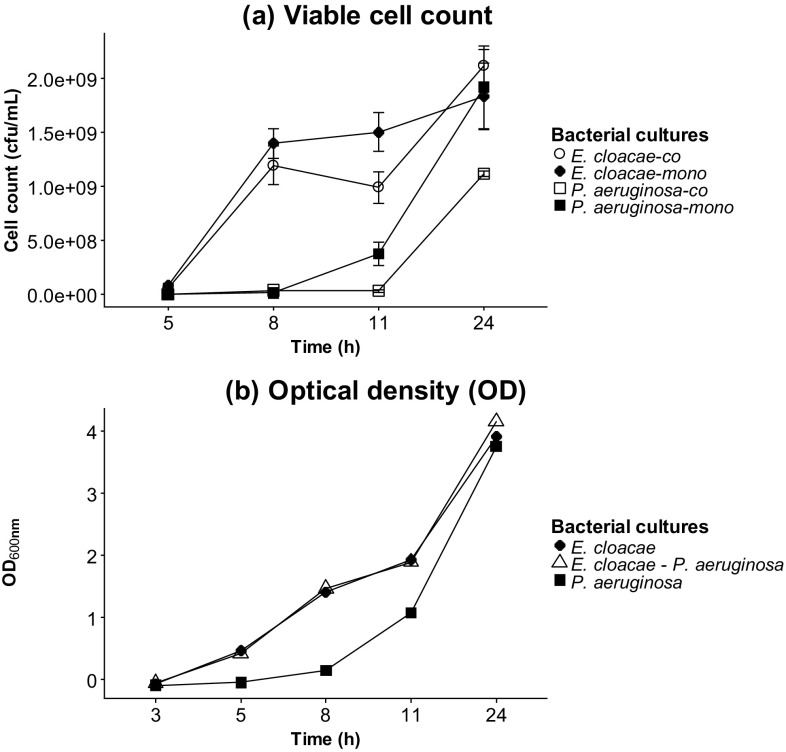
**a** Viable cell counts expressed as colony forming units per mL (cfu/mL), **b** optical densities of mono- and co-cultures of *E. cloacae* and *P. aeruginosa* over a period of 24 h. Viable cell counts were performed in triplicates and the error bars represent standard deviations. Levine EMB agar and artificial sputum were used as solid and liquid media respectively

*E. cloacae* and *P. aeruginosa* were grown individually and together. To determine viable cell numbers, a serial dilution of individual cultures was performed and then plated on Levine EMB agar. *E. cloacae* mono- and co-cultures were observed to have a similar growth pattern and enter the log phase quicker than *P. aeruginosa* which displayed a prolonged lag period especially in the co-culture before entering the growth phase (Fig. 2a). Optical densities were also measured to complement the viable cell counts (Fig. 2b).

### VOC profiling in bacterial cultures

**Fig. 3:**
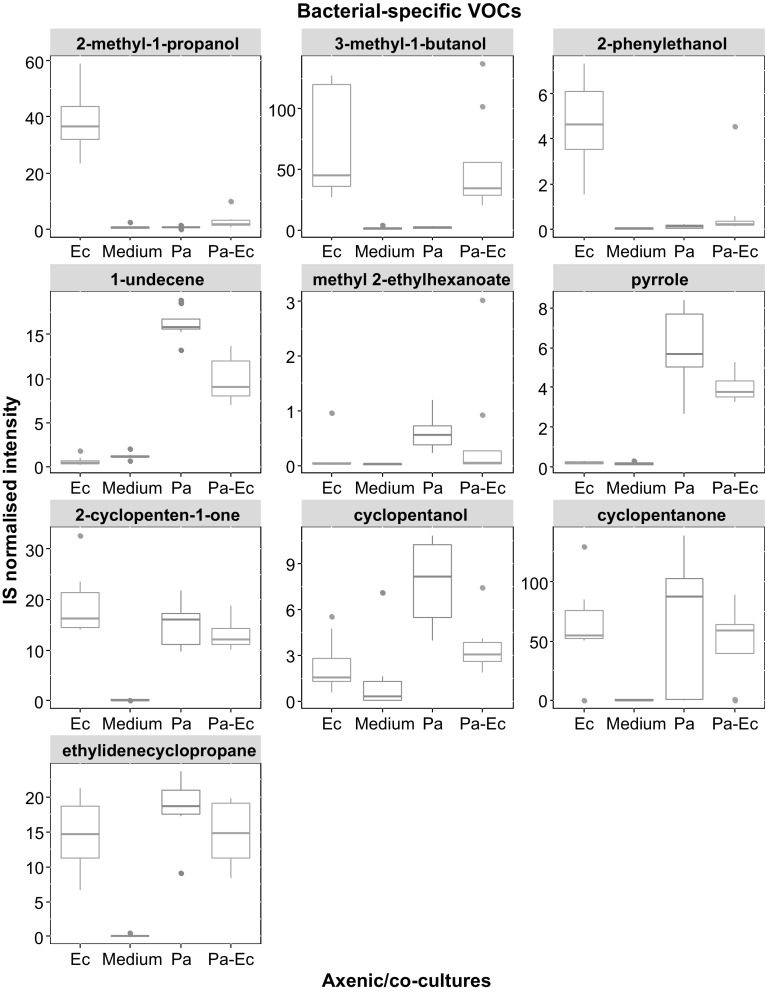
Boxplots showing the internal standard (IS) normalised concentrations of bacteria-specific VOCs in the medium blank, *E. cloacae* (Ec), *P. aeruginosa* (Pa), and *P. aeruginosa–E. cloacae* (Pa–Ec) bacterial cultures. The boxplots were generated from eight repeats

**Fig. 4:**
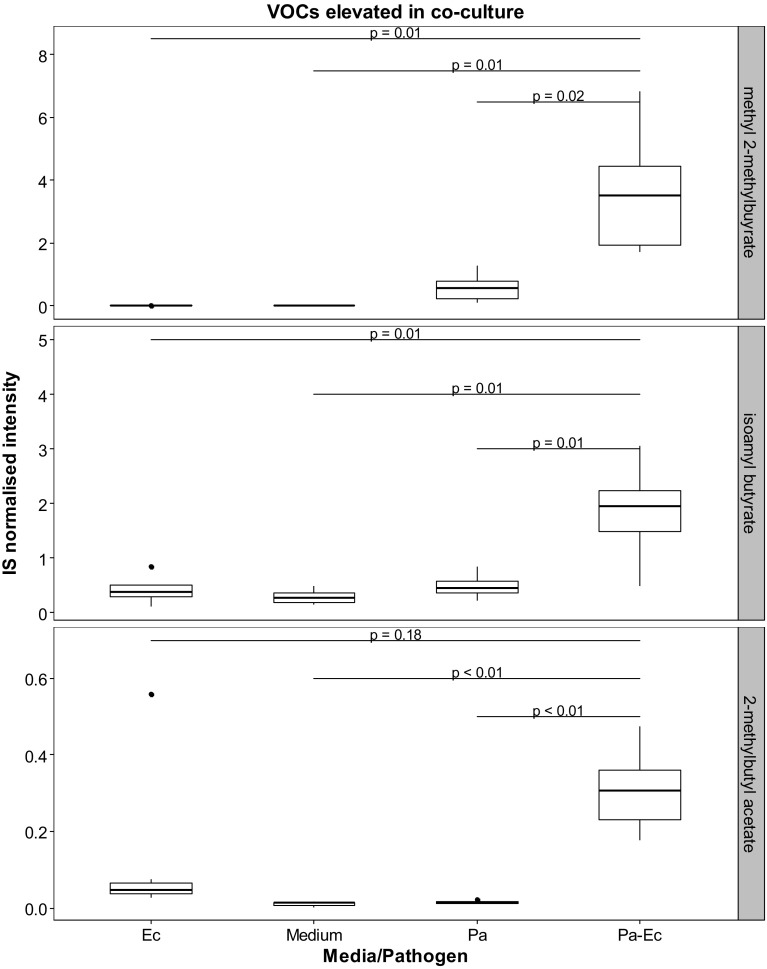
Boxplots showing the internal standard (IS) normalised concentrations of methyl 2-methylbutyrate, isoamyl butyrate, and 2-methylbutyl acetate in headspace of blank medium, *E. cloacae* (Ec), *P. aeruginosa* (Pa), and *P. aeruginosa–E. cloacae* (Pa–Ec) bacterial cultures. These VOCs were found to be elevated in the co-culture samples in comparison to axenic cultures and blank medium. After performing Kruskal–Wallis test, Games-Howell test was used for post-hoc analyses and  *p*-values were corrected by false discovery rate. The boxplots were generated from eight repeats

VOCs originating specifically from bacterial cultures (absent in medium control) are shown in Fig. 3. Metabolites that were found to be present in both the headspace of bacterial cultures and blank medium but at levels that are significantly different between the two after performing post-hoc analyses with correction by false discovery rate are shown in Table 1. Hypothesis testing was performed to compare the concentrations of the VOCs in the headspace of bacterial cultures and medium control and differences indicate whether the VOC was released or consumed. Other identified VOCs are shown in Table 2. VOCs that were observed to be elevated in the bacterial co-culture headspace are shown in Fig. 4. These are putatively identified compounds based on fragmentation pattern comparison to the NIST reference library and are therefore MSI level 2 (Sumner et al. 2007). Representative chromatograms for axenic cultures and co-cultures are shown in the supplementary material (Fig S1).

**Table 1:** VOCs detected in bacterial cultures and blank medium control is shown

VOC	CAS#	*m*/*z*	Ec	Pa	Pa–Ec	Class
2-Ethyl-*trans*-2-butenal	63883-69-2	98 (M^+^)	↓	↓	↓	Aldehyde
Benzaldehyde	100-52-7	77 (bp)	↓	↓	↓	
2-Methylbutanal	96-17-3	41 (bp)	ns	↓	↓	
Furfural	98-01-1	96 (bp) (M^+^)	ns	↓	↓	
Hexanal	66-25-1	44 (bp)	↓	↓	↓	
2-Ethylhexenal	645-62-5	126 (M^+^)	↓	↓	↓	
2-Methylpropanal	78-84-2	43 (bp)	↓	↓	↓	
2,2-Dimethylpropanal	630-19-3	86 (M^+^)	↓	↓	ns	
1-Butanol	71-36-3	56 (bp)	↓	↓	ns	Alcohol
2-Butanol	78-92-2	45 (bp)	ns	↑	ns	
2-Furanmethanol	98-00-0	98 (bp)	↓	↓	↓	
2-Propanol	67-63-0	43 (fg)	ns	↑	ns	
Ethanol	64-17-5	31 (bp)	↓	↓	↓	
Pentane	109-66-0	43 (bp)	↓	↓	↓	Alkane
Ethyl propionate	105-37-3	102 (M^+^)	↑	↓	↑	Ester
2-Methylfuran	534-22-5	82 (bp)	↑	ns	↑	Heterocyclic
Acetone	67-64-1	43 (bp)	↑	↑	↑	Ketone
2-Heptanone	110-43-0	58 (fg)	↑	ns	ns	
2,6-Dimethyl pyrazine	108-50-9	108 (bp) (M^+^)	↑	ns	↑	Nitrogen-containing compound
Dimethyl sulfide	75-18-3	62 (bp)	↑	ns	ns	Sulfur-containing compound
Dimethyl disulfide	624-92-0	94 (bp)	↑	↑	↑	
Methylthioacetate	1534-08-3	90 (fg)	↑	ns	↑	

*(M*^+^*)* molecular ion, *bp* base peak, *fg* fragment

Unless depicted with ns (non-significant), (↑) and (↓) assigned to bacterial cultures indicate a statistically significant (after false discovery rate correction) increase and decrease respectively in headspace concentration in comparison to medium control. The normalised abundances of representative fragments (*m*/*z*) were used for hypothesis testing

**Table 2:** Other (statistically non-significant) VOCs detected in the headspace of bacterial cultures with respect to blank medium control

VOC	CAS#	*m*/*z*	Class
2-Butenal	4170-30-3	41 (bp)	Aldehyde
Acetaldehyde	75-07-0	29 (bp)	
Methacrolein	78-85-3	41 (bp)	
Pentanal	110-62-3	44 (bp)	
2-Ethyl-1-hexanol	104-76-7	55 (fg)	Alcohol
Dodecane	112-40-3	57 (bp)	Alkane
2,4-Dimethylheptane	2213-23-2	43 (bp)	
2,3,5-Trimethylhexane	1069-53-0	43 (bp)	
Nonane	111-84-2	43 (bp)	
4-Methylheptane	589-53-7	43 (bp)	
2,3-Dimethylbutane	79-29-8	43 (bp)	
Decane	124-18-5	57 (bp)	
Heptane	142-82-5	43 (bp)	
Octane	111-65-9	43 (bp)	
2-Methylpentane	107-83-5	43 (bp)	
2,4-Dimethylhexane	589-43-5	43 (bp)	
1,3-Dimethylbenzene	108-38-3	91 (bp)	Aromatic hydrocarbon
1,2,3-Trimethylbenzene	526-73-8	105 (bp)	
Ethylbenzene	100-41-4	91 (bp)	
m-Di-tert-butylbenzene	1014-60-4	175 (bp)	
Benzene	71-43-2	78 (bp) (M^+^)	
α-Pinene	80-56-8	93 (bp)	Alkene
2-Butene	107-01-7	41 (bp)	
2-Methyl-1-pentene	763-29-1	56 (bp)	
2,4-Dimethyl-1-heptene	19549-87-2	43 (bp)	
Ethyl tert-butyl ether	637-92-3	59 (bp)	Ether
2-Ethylfuran	3208-16-0	81 (bp)	Heterocyclic
2,4-Dimethylfuran	3710-43-8	96 (bp) (M^+^)	
2-Pentylfuran	3777-69-3	81 (bp)	
Furan	110-00-9	68 (bp) (M^+^)	
2-Butanone	78-93-3	43 (bp)	Ketone
2-Pentanone	107-87-9	43 (bp)	
3-Hexen-2-one	763-93-9	83 (bp)	
2-Methylthiophene	554-14-3	97 (bp)	Sulfur-containing compound
Carbon disulfide	75-15-0	76 (bp)	
Dimethyl trisulfide	3658-80-8	126 (bp) (M^+^)	
Thiophene	110-02-1	84 (bp) (M^+^)	

The normalised concentrations of representative fragments (*m*/*z*) were used for hypothesis testing

*(M*^+^*)* molecular ion, *bp* base peak, *fg* fragment

### Multivariate analysis

**Fig. 5:**
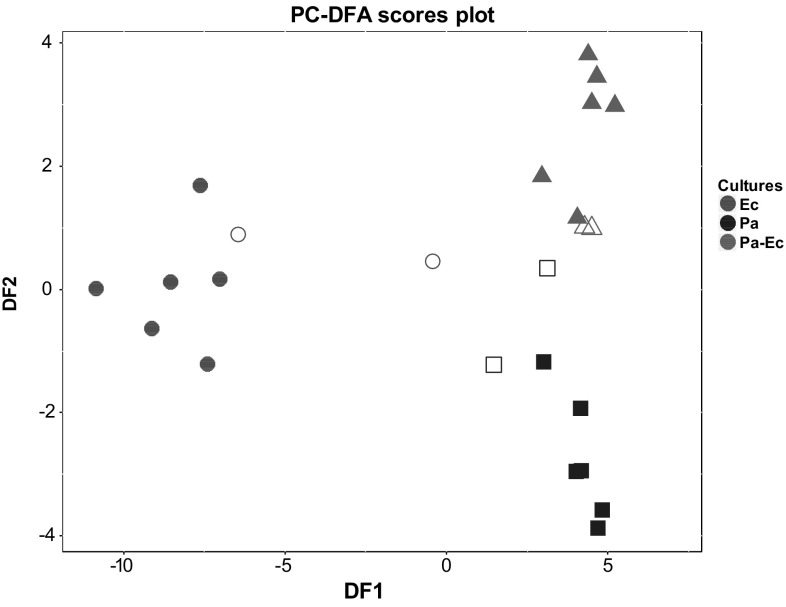
PC–DFA scores validation biplot. For DFA this used 8 PCs (accounting for 64.5% of total explained variance) along with the information on the three classes used as the a priori information. Training samples from the distinct bacterial cultures are indicated as filled shapes while the projected test samples are hollow. *Ec E. cloacae* (circle), *Pa P. aeruginosa* (square), *Pa–Ec P. aeruginosa–E. cloacae* (triangle)

PC–DFA was implemented to visualise the data in dimensional space and discern VOCs that discriminate between respective mono- and co-cultures. Eight PCs were included for DFA as it achieved the lowest root mean square error after performing cross validation. This proportion accounted for approximately 64.5% of conserved variance extracted from the dataset (Fig S2). The training set was used to build the model and the test set to evaluate its validity through projection into subspace established by the training set. The test data were congruent with their respective training set clusters (Fig. 5). From the loadings plot (Fig S3), fragments from 2-methyl-1-propanol and 1-undecene were observed to contribute to the separation between *E. cloacae* mono-culture and *P. aeruginosa* mono- and co-cultures along DF1. Along DF2, the co-culture appears to be separated from both mono-cultures with fragments emanating from 3-methyl-1-butanol, isoamyl butyrate, and 2-methylbutyl acetate.

## Discussion

The viable cell count indicated maximal growth of both microbes after 11 h. Several VOCs were observed in the headspace of axenic cultures and co-cultures. Some metabolites (i.e. 3-methyl-1-butanol, 1-undecene) from axenic cultures were also present in the co-culture headspace while others appear diminished (2-methyl-1-propanol, 2-phenylethanol). Furthermore, some VOCs with very low levels in the mono-cultures were upregulated in the co-culture headspace (methyl 2-methylbutyrate, isoamyl butyrate, and 2-methylbutyl acetate).

The growth dynamic in both mono- and co-cultures of the bacteria was explored by a serial dilution plating method. Levine EMB agar, a selective (allows growth of Gram-negative and suppresses the growth of Gram-positive bacteria) and differential (distinct appearance of colonies based on metabolism of different nutrients) medium was used to discriminate bacterial colonies (Leininger et al. 2001; Parisi and Marsik 1969; Gehm and Heukelekian 1935). In this case *E. cloacae* is capable of metabolising lactose into lactic acid which reduces the pH of the medium thus resulting in the appearance of dark pink colonies (Leininger et al. 2001). In contrast, *P. aeruginosa* is incapable of conducting the aforementioned conversion and instead metabolises peptone which increases the pH of the medium and appear as colourless/white colonies. The observed distinct phenotypes aided in distinguishing the bacterial species for growth monitoring.

The reporting of volatile metabolites originating from *E. cloacae* is scarce in literature. VOCs such as 2-methyl-1-propanol, 3-methyl-1-butanol, and 2-phenylethanol were observed to be specific for this microbe in this study. These metabolites have been reported to be released by other VAP-associated bacterial species (Tait et al. 2014; Filipiak et al. 2012; Junger et al. 2012) thus the use of a sole marker may not be suitable and may require a broad range of selected metabolites to aid microbial identification. VOCs emitted by *P. aeruginosa* have been previously communicated which include 1-undecene and pyrrole which were also observed in this investigation (Filipiak et al. 2012). Methyl 2-ethylhexanoate has not previously been reported. Bacteria specific VOCs such as cyclopentanone and cyclopentanol emitted by both microbes were also observed and have been reported (Lawal et al. 2017b).

In addition to the metabolites measured only in bacterial cultures, some VOCs were observed in both blank medium and bacterial cultures and may be useful. The statistically significant compounds can be separated from the redundant components and may potentially prove to be a reliable addition to bacteria-specific VOCs when compiling a panel of biomarkers for microbial identification. Other sterile media VOCs especially aldehydes were observed to be consumed. This consumption has previously being reported for *P. aeruginosa* (Filipiak et al. 2012). This phenomenon was also observed for alcohols except for 2-butanol which appears to be released by *P. aeruginosa*.

Also novel to our study was the investigation of potential markers for co-habitation of two pathogenic microbes. Commensal microbes are ubiquitously distributed in humans and have an important role in limiting the influence of ‘foreign’ potentially pathogenic microflora (Martin et al. 2013; Littman and Pamer 2011; Ubeda et al. 2017). *E. cloacae* is found in the environment and also known to be a member of the gut flora of a healthy host and is considered a commensal microbe (Keller et al. 1998; Davin-Regli and Pages 2015). However, it is implicated as an opportunistic pathogen in VAP and evidence suggests that the endogenous reservoir of this microbe is the predominant source as a result of translocation from the gut to the lung (Keller et al. 1998; Park 2005). Therefore in conjunction with opportunistic pathogens such as *P. aeruginosa* it may culminate in a poly-microbial infection in a host. *E. cloacae* appears to enter the growth phase in co-culture in a shorter period (after 5 h) in comparison to *P. aeruginosa* which needed approximately 11 h before entering the log phase. This prolonged lag phase may be as a result of a larger genome size in comparison to *E. cloacae* (Ren et al. 2010; Stover et al. 2000). After a 24 h incubation period of the bacterial combination, some VOCs were observed to be elevated in co-culture. Methyl 2-methylbutyrate appears to be produced by *P. aeruginosa* and has been previously reported (Filipiak et al. 2012) but is elevated in co-culture. Similarly, 2-methylbutyl acetate appears to be produced by *E. cloacae* and increased levels was observed in co-culture. Overexpression of indole by *E. coli* when in co-culture with *P. aeruginosa* has been observed and shown to be a virulence factor which suppresses growth of *P. aeruginosa* (Chu et al. 2012; Culotti and Packman 2014). Thus it could be the case that these metabolites are overexpressed in an attempt to induce suppression and establish dominance, although at this stage there is no direct evidence of this. Both compounds have been reported to exhibit antimicrobial activity in a study investigating the antimicrobial properties of Roman chamomile oil (Bail et al. 2009). 2-methylbutyl acetate displays antimicrobial activity towards a variety of microbes including *P. aeruginosa*, and thus its upregulation by *E. cloacae* may be a strategy to deter the growth of the former and may have also contributed to the delayed lag phase observed from the viable cell counts. As suppression of *P. aeruginosa* was not achieved, this may have provided the opportunity for this bacterium to reciprocate by emitting its own antimicrobial compound methyl 2-methylbutyrate. This metabolite is reported to be ineffective against *E. coli* and *K. pneumoniae* (Bail et al. 2009) and therefore may not be effective against *E. cloacae* since they belong to the same family. It has also been previously reported that the metabolism of a co-cultured species changes under detection of emitted VOCs by another species (Dow 2017). It would be interesting to investigate beyond 24 h to understand and gain insight into this relationship.

PC–DFA was used to visualise the relationship between the mono-cultures along with the co-culture of both bacteria. PC–DF1 which accounts for the largest group separation separated *E. cloacae* from *P. aeruginosa* and the second PC–DF allowed some separation of the co-culture from *P. aeruginosa*, although it was clear that the VOCs of the co-culture was more similar to *P. aeruginosa* than it was to *E. cloacae*. VOCs found in mono- (2-methyl-1propanol, 3-methyl-1-butanol, and 1-undecene) and co-cultures (isoamyl butyrate and 2-methylbutyl acetate) were observed to be the main contributors to the separation observed along DF1 and DF2 axis of the PC–DFA scores plot.

These metabolites can be sought after in breath specimens obtained from patients and have their biomarker credentials assessed. Another potential application is to screen these compounds from results of headspace analysis of lower respiratory tract specimens obtained from patients. Although still an invasive procedure, it would eliminate the delay associated with pathogen identification and aid targeted antibiotic therapy.

## Conclusion

VOCs in exhaled breath have potential for clinical translation into the clinic as proven in the case of exhaled nitric oxide used in the diagnosis of asthma. To extend this application for infection diagnosis, microbial VOCs originating from *E. cloacae* and *P. aeruginosa* mono- and co-cultures were investigated in vitro. We observed VOCs such as 2-methyl-1-propanol, 2-phenylethanol, 1-undecene and pyrrole in axenic cultures that can be utilised for bacterial species differentiation. In addition, VOCs including 2-methylbutyl acetate and methyl 2-methylbutyrate can be useful in discriminating mono- and co-cultures which may potentially translate to the distinction between mono- and poly-microbial infections. Validation of these in vitro markers in breath specimens or airway samples can potentially prove to be a clinically useful tool for elucidating causal pathogen identification and infection type discernment which may translate into appropriate antibiotic therapy regimen and improve patient outcomes.

## Electronic supplementary material

Below is the link to the electronic supplementary material.

Supplementary material 1 (DOCX 423 KB)

## Data Availability

The data in this study are deposited in MetaboLights (https://www.ebi.ac.uk/metabolights/) study identifier MTBLS617.

## References

[CR1] Ahmed WM, lawal O, Nijsen TM, Goodacre R, Fowler SJ (2017). Exhaled volatile organic compounds of infection: A systematic review. ACS Infectious Diseases.

[CR2] Allardyce RA, Langford VS, Hill AL, Murdoch DR (2006). Detection of volatile metabolites produced by bacterial growth in blood culture media by selected ion flow tube mass spectrometry (SIFT-MS). Journal of Microbiological Methods.

[CR3] Bail S, Buchbauer G, Jirovetz L, Denkova Z, Slavchev A, Stoyanova A, Schmidt E, Geissler M (2009). Antimicrobial activities of roman chamomile oil from France and its main compounds. Journal of Essential Oil Research.

[CR4] Boots AW, Smolinska A, van Berkel JJ, Fijten RR, Stobberingh EE, Boumans ML, Moonen EJ, Wouters EF, Dallinga JW, van Schooten FJ (2014). Identification of microorganisms based on headspace analysis of volatile organic compounds by gas chromatography-mass spectrometry. Journal of Breath Research.

[CR5] Chu W, Zere TR, Weber MM, Wood TK, Whiteley M, Hidalgo-Romano B, Valenzuela E, Mclean RJ (2012). Indole production promotes *Escherichia Coli* mixed-culture growth with *Pseudomonas aeruginosa* by inhibiting quorum signaling. Applied and Environment Microbiology.

[CR6] Combes A, Figliolini C, Trouillet JL, Kassis N, Wolff M, Gibert C, Chastre J (2002). Incidence and outcome of polymicrobial ventilator-associated pneumonia. Chest.

[CR7] Culotti A, Packman AI (2014). *Pseudomonas aeruginosa* Promotes *Escherichia Coli* Biofilm Formation in Nutrient-Limited Medium. PLoS ONE.

[CR8] Davin-Regli A, Pages JM (2015). *Enterobacter aerogenes* and *Enterobacter cloacae*; versatile bacterial pathogens confronting antibiotic treatment. Frontiers in Microbiology.

[CR9] Diraviam Dinesh S (2010). Artificial sputum medium. Protocol Exchange.

[CR10] Dow JM (2017). Diffusible signal factor-dependent quorum sensing in pathogenic bacteria and its exploitation for disease control. Journal of Applied Microbiology.

[CR11] Ferrer M, Difrancesco LF, Liapikou A, Rinaudo M, Carbonara M, Li Bassi G, Gabarrus A, Torres A (2015). Polymicrobial intensive care unit-acquired pneumonia: Prevalence, microbiology and outcome. Critical Care.

[CR12] Filipiak W, Sponring A, Baur MM, Filipiak A, Ager C, Wiesenhofer H, Nagl M, Troppmair J, Amann A (2012). Molecular analysis of volatile metabolites released specifically by *Staphylococcus aureus* and *Pseudomonas aeruginosa*. BMC Microbiology.

[CR13] Gehm HW, Heukelekian H (1935). Eosin methylene blue agar for rapid direct count of *E. coli*. American Journal of Public Health and the Nations Health.

[CR14] Goodacre R, Timmins EM, Burton R, Kaderbhai N, Woodward AM, Kell DB, Rooney PJ (1998). Rapid identification of urinary tract infection bacteria using hyperspectral whole-organism fingerprinting and artificial neural networks. Microbiology.

[CR15] John JF, Sharbaugh RJ, Bannister ER (1982). *Enterobacter cloacae*: Bacteremia, epidemiology, and antibiotic resistance. Reviews of Infectious Diseases.

[CR16] Jombart T (2008). Adegenet: A R package for the multivariate analysis of genetic markers. Bioinformatics.

[CR17] Jombart T, Devillard S, Balloux F (2010). Discriminant analysis of principal components: A new method for the analysis of genetically structured populations. BMC Genetics.

[CR18] Junger M, Vautz W, Kuhns M, Hofmann L, Ulbricht S, Baumbach JI, Quintel M, Perl T (2012). Ion mobility spectrometry for microbial volatile organic compounds: A new identification tool for human pathogenic bacteria. Applied Microbiology and Biotechnology.

[CR19] Kalanuria AA, Ziai W, Mirski M (2014). Ventilator-associated pneumonia in the ICU. Critical Care.

[CR20] Keller R, Pedroso MZ, Ritchmann R, Silva RM (1998). Occurrence of virulence-associated properties in *Enterobacter cloacae*. Infection and Immunity.

[CR21] Lawal O, Ahmed WM, Nijsen TME, Goodacre R, Fowler SJ (2017). Exhaled breath analysis: A review of ‘breath-taking’ methods for off-line analysis. Metabolomics.

[CR22] Lawal O, Muhamadali H, Ahmed W, White IR, Nijsen TME, Goodacre R, Fowler SJ (2017). Headspace volatile organic compounds from bacteria implicated in ventilator-associated pneumonia analysed by TD-GC/MS. Journal of Breath Research.

[CR23] Leininger DJ, Roberson JR, Elvinger F (2001). Use of eosin methylene blue agar to differentiate *Escherichia Coli* from other gram-negative mastitis pathogens. Journal of Veterinary Diagnostic Investigation.

[CR24] Littman DR, Pamer EG (2011). Role of the commensal microbiota in normal and pathogenic host immune responses. Cell Host & Microbe.

[CR25] Lourenco C, Turner C (2014). Breath analysis in disease diagnosis: Methodological considerations and applications. Metabolites.

[CR26] Martin R, Miquel S, Ulmer J, Kechaou N, Langella P, Bermudez-Humaran LG (2013). Role of commensal and probiotic bacteria in human health: A focus on inflammatory bowel disease. Microbial Cell Factories.

[CR27] Mietto C, Pinciroli R, Patel N, Berra L (2013). Ventilator associated pneumonia: Evolving definitions and preventive strategies. Respiratory Care.

[CR28] Neerincx AH, Geurts BP, Habets MF, Booij JA, Van Loon J, Jansen JJ, Buydens LM, Van Ingen J, Mouton JW, Harren FJ, Wevers RA, Merkus PJ, Cristescu SM, Kluijtmans LA (2016). Identification of *Pseudomonas aeruginosa* and *Aspergillus fumigatus* mono- and co-cultures based on volatile biomarker combinations. Journal of Breath Research.

[CR29] Parisi JT, Marsik FJ (1969). Atypical reactions of *Escherichia Coli* on eosin methylene blue Agar. Applied Microbiology.

[CR30] Park DR (2005). The microbiology of ventilator-associated pneumonia. Respiratory Care.

[CR31] Phillips M (1992). Breath tests in medicine. Scientific American.

[CR32] Ren Y, Ren Y, Zhou Z, Guo X, Li Y, Feng L, Wang L (2010). Complete genome sequence of *Enterobacter cloacae* subsp. cloacae type strain ATCC 13047. Journal of Bacteriology.

[CR33] Shestivska V, Nemec A, Drevinek P, Sovova K, Dryahina K, Spanel P (2011). Quantification of methyl thiocyanate in the headspace of *Pseudomonas aeruginosa* cultures and in the breath of cystic fibrosis patients by selected ion flow tube mass spectrometry. Rapid Communications in Mass Spectrometry.

[CR34] Smith CA, Want EJ, O’maille G, Abagyan R, Siuzdak G (2006). XCMS: Processing mass spectrometry data for metabolite profiling using nonlinear peak alignment, matching, and identification. Analytical Chemistry.

[CR35] Stover CK, Pham XQ, Erwin AL, Mizoguchi SD, Warrener P, Hickey MJ, Brinkman FS, Hufnagle WO, Kowalik DJ, Lagrou M, Garber RL, Goltry L, Tolentino E, Westbrock-Wadman S, Yuan Y, Brody LL, Coulter SN, Folger KR, Kas A, Larbig K, Lim R, Smith K, Spencer D, Wong GK, Wu Z, Paulsen IT, Reizer J, Saier MH, Hancock RE, Lory S, Olson MV (2000). Complete genome sequence of *Pseudomonas aeruginosa* PAO1, an opportunistic pathogen. Nature.

[CR100] Sumner LW, Amberg A, Barrett D, Beale MH, Beger R, Daykin CA, Fan TW, Fiehn O, Goodacre R, Griffin JL, Hankemeier T, Hardy N, Harnly J, Higashi R, Kopka J, Lane AN, Lindon JC, Marriott P, Nicholls AW, Reily MD, Thaden JJ, Viant MR (2007). Proposed minimum reporting standards for chemical analysis: Chemical Analysis Working Group (CAWG) Metabolomics Standards Initiative (MSI). Metabolomics.

[CR36] Tait E, Perry JD, Stanforth SP, Dean JR (2014). Identification of volatile organic compounds produced by bacteria using HS-SPME-GC-MS. Journal of Chromatographic Science.

[CR37] Ubeda C, Djukovic A, Isaac S (2017). Roles of the intestinal microbiota in pathogen protection. Clinical and Translational Immunology.

[CR38] Zhu J, Bean HD, Kuo YM, Hill JE (2010). Fast detection of volatile organic compounds from bacterial cultures by secondary electrospray ionization-mass spectrometry. Journal of Clinical Microbiology.

[CR39] Zscheppank C, Wiegand HL, Lenzen C, Wingender J, Telgheder U (2014). Investigation of volatile metabolites during growth of *Escherichia Coli* and *Pseudomonas aeruginosa* by needle trap-GC-MS. Analytical and Bioanalytical Chemistry.

