# Supplementary material for: TD/GC–MS analysis of volatile markers emitted from mono- and co-cultures of Enterobacter cloacae and Pseudomonas aeruginosa in artificial sputum
Source: Metabolomics. 2018 Apr 26;14(5):66. doi: 10.1007/s11306-018-1357-5 (PMC5920131; doi:10.1007/s11306-018-1357-5)
Supplement: Supplementary file 1 — Supplementary material 1 (DOCX 423 KB) [file 11306_2018_1357_MOESM1_ESM.docx]

**TD/GC-MS analysis of volatile markers emitted from mono- and co-cultures of *Enterobacter cloacae* and *Pseudomonas aeruginosa* in artificial sputum**

Oluwasola Lawal^1,4^, Hugo Knobel^3^, Hans Weda^2^, Tamara M.E. Nijsen^2^, Royston Goodacre^4^, Stephen J. Fowler^1,5^ on behalf of the BreathDx consortium

^1^Division of Infection, Immunity and Respiratory Medicine, School of Biological Sciences, Faculty of Biology, Medicine and Health, The University of Manchester, Manchester, United Kingdom

^2^Philips Research, Royal Philips B.V., Eindhoven, The Netherlands

^3^Philips Innovation Labs, Philips Lighting, Eindhoven, The Netherlands

^4^School of Chemistry, Manchester Institute of Biotechnology, University of Manchester, United Kingdom

^5^Manchester Academic Health Science Centre, The University of Manchester and Manchester University NHS Foundation Trust, Manchester, United Kingdom

Supplementary information

**Chromatograms**

Representative chromatograms for *E. cloacae* and *P. aeruginosa* mono- and co-culture bacterial samples are shown below (Fig S1).

**Fig S1.** Representative chromatograms of *E. cloacae* and *P. aeruginosa* axenic cultures and co-culture.

**Principal component (PC) tuning**

Principal component analysis (PCA) was performed on X block data (VOC profile) to evaluate the number of PCs to input for DFA. Eight PCs were selected which accounts for approximately 64.5% of variance in the dataset (Fig S2).

**Fig S2.** Scree plot of PCA indicating the number of retained principal components (PCs) extracted and the corresponding cumulative explained variance.

**PC-DFA loadings plot**

The loadings plots from PC-DFA analysis are shown in Fig S3. The fragments at the extremity of the loading plots were investigated as they are major contributors to the observed separation in the scores plot (Fig 5). Fragments 90, 465, & 2046 belong to 2-methyl-1-propanol and fragments 577, 859 to 1-undecene (Fig S3a). The identity of the other fragments is still unknown. In the loadings plot of DF2, fragment 1083 originates from 3-methyl-1-butanol, 2012 from 2-methylbutyl acetate, and 1877 from isomayl butyrate.

**Fig S3.** PC-DFA loadings plot for (a) DF1, (b) DF2.
